# Development Track of Compliance With Dual Antiplatelet Therapy and Analysis of Potential Categories in Patients With ACS After PCI

**DOI:** 10.31083/RCM37270

**Published:** 2025-08-19

**Authors:** Xiaoqing Zheng, Ya Wang, Hongxing Wang, Minfang Guan, Xiaosong Chen, Shasha Li, Wanluan Zhang, Jiamiao Hu, Qiaoling Ye, Qinhong Xu

**Affiliations:** ^1^Cardiovascular Internal Medicine Ward, The First Affiliated Hospital of Ningbo University, 315000 Ningbo, Zhejiang, China; ^2^Cardiovascular Internal Medicine Ward, The Yangming Hospital Affiliated to Ningbo University, 315400 Yuyao, Zhejiang, China; ^3^Cardiovascular Internal Medicine Ward, Cixi Longshan Hospital, 315300 Cixi, Zhejiang, China; ^4^Nursing Department, The First Affiliated Hospital of Ningbo University, 315000 Ningbo, Zhejiang, China

**Keywords:** percutaneous coronary intervention, dual anti-platelet therapy, growth mixture modeling (GMM), development track, potential categories

## Abstract

**Background:**

To explore the potential categories of compliance development track of dual antiplatelet therapy (DAPT) after percutaneous coronary intervention (PCI) in patients with acute coronary syndrome (ACS) using growth mixture modeling (GMM) to analyze its predictive factors, providing evidence for dynamic adherence monitoring and tailored interventions.

**Methods:**

A total of 150 patients with ACS after PCI were selected by convenience sampling. Patients were studied using Self-Efficacy for Appropriate Medication Use Scale (SEAMS), family APGAR index (APGAR), Generalized Anxiety Disorder-2 (GAD-2), and Patient Health Questionnaire-2 (PHQ-2) at baseline. The compliance of patients with DAPT was assessed using Morisky Medication Adherence Scales-8 (MMAS-8) at 1, 3, 6, 9, and 12 months after discharge. The mixed model of latent variable growth was used to identify the development track of compliance. Multiple logistic regression was used to analyze the predictive factors of different development track categories.

**Results:**

Two development track categories of DAPT compliance in patients with ACS after PCI were identified in the low compliance-decreased group (7.41%) and the persistent high compliance group (92.59%). Multivariate logistic regression analysis showed that age ≥60 years, body mass index (BMI), and the family APGAR index were the predictive factors of different development track categories of DAPT compliance in patients with ACS after PCI.

**Conclusion:**

Significant population heterogeneity was observed in the development track of DAPT in ACS patients within 12 months after PCI. The compliance of most patients remained stable, and only a few remained at a low level and showed a significant downward trend. Based on these predictive factors, healthcare personnel can identify patients in the low compliance–decreased group early and implement targeted and specific interventions to improve DAPT compliance of ACS patients after PCI.

## 1. Introduction

According to the global disease burden study [1], there were approximately 523 million cardiovascular disease cases and 18.6 million cardiovascular disease deaths worldwide in 2019; these are the main causes of the disease burden globally. Acute coronary syndrome (ACS) is a common and severe coronary heart disease. ACS refers to the symptoms caused by coronary artery obstruction [2]. In 2020, the mortality rate of ACS in urban areas was 60.29/100,000, and that of ACS in rural areas was 78.65/100,000 [3]. Percutaneous coronary intervention (PCI) is the most common vascular reconstruction method for patients with ACS. The annual cases of PCI show an increasing trend. The number of interventional cases in mainland China in 2021 reached 1,164,117, averaging 1.48 implanted stents [3]. Dual antiplatelet therapy (DAPT), comprising aspirin and P2Y12 inhibitors, is an important tool for treating and preventing atherosclerotic events. Six to twelve months of DAPT after PCI helps reduce the risk of adverse cardiovascular events. DAPT is the cornerstone of drug treatment for patients with ACS after PCI [4]. A previous study demonstrated that adherence to antiplatelet drugs in 4.5% of patients with coronary heart disease exhibited a slow decline [5]. Non-adherence to DAPT remains a critical challenge in post-PCI management. DAPT treatment evolves into dynamic changes with time. After PCI, patients interrupt DAPT treatment due to non-compliance. The common interruption factors are older age, Canadian Cardiovascular Society (CCS) class II angina pectoris, living in rural areas, education level, marital status, time from PCI, hypertension, and the number of complications [6, 7, 8]. However, current evidence predominantly relies on cross-sectional designs or single-time-point adherence assessments. There remains a lack of research on DAPT compliance in patients after PCI.

Traditional approaches dichotomize adherence as “high” or “low”, overlooking temporal heterogeneity within populations. Growth mixture modeling (GMM) can analyze trends of longitudinal data and explain the differences in individual changes within different development track categories. GMM is often used to identify the heterogeneous development track of health behaviors of patients with chronic diseases. Therefore, this study sought to track and investigate drug compliance of ACS patients with DAPT after PCI, identify the longitudinal development track of compliance using GMM, and analyze the predictive factors between different track categories, to provide a theoretical basis for guiding the use of DAPT in ACS patients after PCI. This would allow medical staff to intervene in advance based on the risk factors affecting DAPT compliance of ACS patients after PCI to improve compliance and reduce the occurrence of adverse cardiac events, which will improve the prognosis and quality of life of patients. We hypothesize that DAPT adherence within 12 months post-PCI will exhibit at least two different developmental trajectories and be influenced by factors such as age, body mass index (BMI), and family support.

## 2. Methods

### 2.1 Participants

Using the convenience sampling method, we selected inpatients diagnosed with ACS and undergoing PCI in the heart center from June 2022 to March 2023. Inclusion criteria included: (1) age ≥18 years old; (2) a diagnosis of ACS undergoing PCI; (3) heart function class I–III, left ventricular ejection fraction (LVEF) ≥35%; (4) the patients, their accompanying person(s), will use a smartphone; (5) informed consent was obtained for participation in this study. Exclusion criteria included (1) patients with severe physical diseases; (2) patients who were automatically discharged, transferred, or died in the hospital; (3) patients who were participating in other studies; (4) withdrawal from the study during the follow-up period; (5) death or loss of contact during follow-up. The Corporate Ethics Committee approved this study. All the participants voluntarily agreed to participate in the study and signed the informed consent form.

### 2.2 Study Tools

A general information questionnaire that includes age, gender, marital status, past medical history, medication history, surgery, laboratory data, and other related information.

#### 2.2.1 Morisky Medication Adherence Scales-8 (MMAS-8)

We use the Chinese version of the MMAS-8 to assess medication compliance [9, 10]. The answers to items 1–7 on the scale were “yes” and “no”. Except for item 5, the answers to the other items were “yes”, and 0 points were scored. An answer of “no” was 1 point, and item 5 was the opposite. The answers to item 8 were “rarely or never”, “occasionally”, “sometimes”, “often”, and “always”, which scored 1, 0.75, 0.5, 0.25, and 0 points, respectively. The overall score for the scale was 8 points. The Cronbach’s α value for the Chinese version of MMAS-8 was 0.81.

#### 2.2.2 Self-Efficacy for Appropriate Medication Use Scale (SEAMS)

The SEAMS [11] was used to measure the self-efficacy of patients taking medication, with 13 items. For this scale, the subjects rated themselves according to their confidence in administering their prescribed medication. According to the Likert 3-level scoring method, one point indicated “no confidence”, two points indicated “a little confidence”, and three points indicated “very confident”. The higher the score, the higher the self-efficacy of patients regarding medication administration. The Cronbach’s α value for the Chinese version of SEAMS was 0.93.

#### 2.2.3 Family APGAR Index (APGAR)

The APGAR [12] is a subjective quantitative evaluation tool for the satisfaction of family members with their families. The APGAR evaluates the satisfaction of five aspects of family function: fitness, cooperation, growth degree, emotional degree, and intimacy degree. There are five items, each with a three-level scoring method of 0–2 points. The Cronbach’s α value was 0.80–0.88.

#### 2.2.4 Generalized Anxiety Disorder-2 (GAD-2)

The GAD-2 [13] can be initially used to screen patients for anxiety symptoms. The total score ranged from 0 to 6 points. The score for each item was as follows: 0 points, “never”; 1 point, “occasionally over a few days”; 2 points, “frequently over a few days” and more than one week in the past two weeks; 3 points, “almost entirely over a few days”. The total score was the sum of the scores for each item. The Cronbach’s α value was 0.867.

#### 2.2.5 Patient Health Questionnaire-2 (PHQ-2)

The PHQ-2 [14] is used to determine whether the subjects were likely to have depression initially. The total score ranges from 0 to 6 points. Those with a score of ≥3 points indicated potential depression. The Cronbach’s α value was 0.84.

### 2.3 Data Collection

Data collection is divided into two parts. In the first part, general data, including SEAMS, APGAR, GAD-2, and PHQ-2 scores of ACS patients, were collected on the day after PCI and before discharge. In the second part, we collected the MMAS-8 scores at 1 month (T1), 3 months (T2), 6 months (T3), 9 months (T4), and 12 months (T5) after PCI for each patient.

### 2.4 Statistical Analysis

The R language was used for data analysis. For descriptive statistics, quantitative data that conform to normal distribution were described as the mean, standard deviation and those that do not conform are described as median and quartile, *t*-test and Mann-Whitney U test were used. Counting data were expressed as frequency (n) and percentage (%), chi-square test, continuous corrected chi-square tests, and fisher’s exact test were used. In modeling the heterogeneous development track, the hlme function in the lcmm package was used to fit the GMM. The number of categories gradually increased with the single-category model until the optimal model (with the smallest Bayesian information criterion (BIC) value) was found. After the track categories were determined, repeated measures of the analysis of variance (ANOVA) were used to compare drug compliance in different categories at different time points. Univariate and multivariate logistic models were used to identify the predictive factors. A value of *p* < 0.05 was defined as statistically significant.

## 3. Results

### 3.1 Participant Patient Profiles

A total of 150 patients undergoing PCI after ACS were included in this study. During the five follow-up visits, a total of 15 cases were lost to follow-up: males (n = 13), aged ≥60 years old (n = 5), high school or above educational background (n = 4), smoking history (n = 9), drinking history (n = 4), diabetes (n = 4), and hypertension (n = 6). No statistical difference was observed in the general information of the included cases. The final sample size was 135 cases (Table 1). There were 108 males (80.0%) and 27 females (20.0%); 90 cases (66.7%) were aged ≥60 years; the rate of medical insurance coverage was 95.6%; 39 cases (28.9%) had an educational status of high school or above. The percentages of patients with a smoking history, drinking history, diabetes, hypertension, and stroke were 45.9%, 30.4%, 24.4%, 54.1%, and 5.9%, respectively. The BMI was 24.52 ± 3.45, and the APGAR index was 10.50 ± 2.94.

**Table 1.** **Demographics and baseline characteristics of the study participants**.

Variable		n (%)	Variable		n (%)
Gender			Occupation		
	Male	108 (80.0%)		Incumbent	44 (32.6%)
	Female	27 (20.0%)		Retired	91 (67.4%)
Age			Daily exercise time		
	<60	45 (33.3%)		<30 min	62 (45.9%)
	≥60	90 (66.7%)		30–60 min	49 (36.3%)
Health insurance situation				>60 min	24 (17.8%)
	Medical insurance	129 (95.6%)	Smoking history		62 (45.9%)
	Self-paid expenses	6 (4.4%)	Drinking history		41 (30.4%)
Marital status			Diabetes		33 (24.4%)
	Married	124 (91.9%)	Hypertension		73 (54.1%)
	Unmarried/divorced/widowed	11 (8.1%)	Stroke		8 (5.9%)
Living status			Medication use		
	Live alone	11 (8.1%)		History of aspirin	101 (74.8%)
	Lives with others	124 (91.9%)		History of ticagrelor	47 (34.8%)
Educational status				History of clopidogrel	52 (38.5%)
	Junior secondary and below	96 (71.1%)		History of statins	102 (75.6%)
	High school and above	39 (28.9%)	Stent number		
LVEF ($\bar{x}$ ± s)		61.02 ± 7.20		<2	60 (44.4%)
BMI ($\bar{x}$ ± s)		24.52 ± 3.45		≥2	75 (55.6%)
SEAMS ($\bar{x}$ ± s)		32.71 ± 5.71	GAD-2		1 (0, 2)
APGAR ($\bar{x}$ ± s)		10.50 ± 2.94	PHQ-2		0 (0, 2)

LVEF, left ventricular ejection fraction; BMI, body mass index; SEAMS, Self-efficacy for Appropriate Medication Use Scale; APGAR, family APGAR index; GAD-2, Generalized Anxiety Disorder-2; PHQ-2, Patient Health Questionnaire-2.

### 3.2 Identification and Determination of Compliance With DAPT in Patients With ACS After PCI

GMM was used in this study to analyze the changing trend in DAPT compliance of ACS patients after PCI, and to identify the heterogeneous development track of ACS patients after PCI. Five DAPT compliance indexes of ACS patients after PCI were fitted, and one to four categories of GMM models were gradually established. The results showed that the model was classified into two groups, with the largest loglik value and the smallest BIC value, so two potential categories were selected for this study (Table 2).

**Table 2.** **Choice of mixed growth model of DAPT compliance in patients with ACS after PCI (n = 135)**.

Number of categories	loglik	BIC	Category probability			
			% Class 1	% Class 2	% Class 3	% Class 4
1	–694.066	1417.564	100			
2	–638.787	1321.722	7.41	92.59		
3	–638.787	1336.438	7.41	92.59	0	
4	–638.787	1351.153	7.41	0	92.59	0

BIC, Bayesian information criterion; ACS, acute coronary syndrome; PCI, percutaneous coronary intervention; DAPT, dual antiplatelet therapy.

We sequentially established GMM models with 1 to 4 classes by fitting dual antiplatelet adherence indices measured at five postoperative time points. The results demonstrated that the two-class model exhibited a higher log-likelihood (LL) value and lower BIC value than the one-class model. In the two-class solution, 7.41% of the population was classified into subgroup 1, while 92.59% comprised subgroup 2. When testing the three-class models, the LL value plateaued with increased BIC, and the third subgroup showed a class probability of 0%. Similarly, the four-class model demonstrated further BIC inflation with two additional zero-probability subgroups. Therefore, both BIC minimization criteria and substantive interpretability supported the optimality of the two-class solution.

**Fig. 1.**
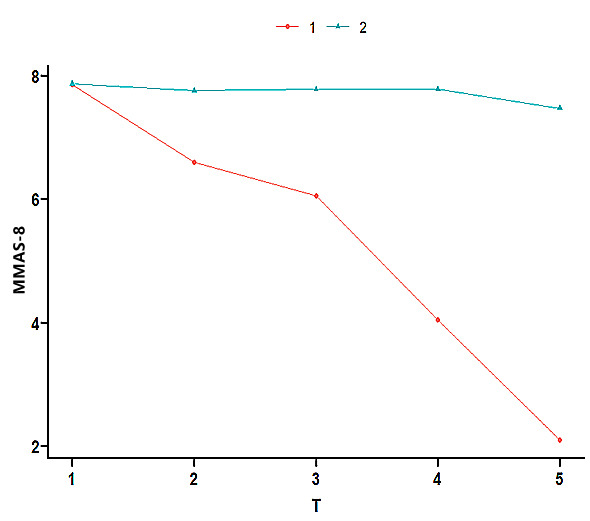
**Development track of the mixed model of latent variable growth in DAPT compliance in patients with ACS after PCI**. TI, T2, T3, T4, and T5 were recorded 1, 3, 6, 9, and 12 months after discharge of the ACS patient after PCI, respectively. MMAS-8, Morisky Medication Adherence Scales-8.

According to the two GMM categories, the heterogeneous development track of ACS patients after PCI was plotted (Fig. 1). There were two completely different developmental features of DAPT compliance in patients with ACS after PCI. A continuous downward trend in MMAS-8 was observed in the first category (low compliance–decreased group). A continuously stable trend in MMAS-8 was noted in the second category (persistently high compliance group).

### 3.3 Comparison of Differences in Compliance Indices of DAPT Among Patients With ACS After PCI

Repeated measures ANOVA showed significant differences in the DAPT compliance of ACS patients after PCI in different groups and at various time points. The F statistic of the interaction terms of time and group was 83.86, and the corresponding *p*-value was less than 0.001, showing statistical significance (Table 3).

**Table 3.** **Repeated measures ANOVA**.

	Freedom	Sum of squares	Mean square	Variance ratio	Pr (>F)
Time	4	14.58	3.65	10.05	<0.001
Group	1	205.05	205.05	565.11	<0.001
Time group	4	121.72	30.43	83.86	<0.001
Residual	660	239.48	0.36		

### 3.4 Analysis of Development Track Category Predictors of DAPT Compliance in Patients With ACS After PCI

Univariate analysis showed that age, occupational status and APGAR index values were statistically significant between the two groups (*p* < 0.05). No significant differences were observed between the two groups in gender, health insurance status, marital status, whether living alone or not, educational level, daily exercise time, smoking history, diabetes history, diabetes, hypertension, stroke, history of aspirin, history of ticagrelor, history of clopidogrel, history of statins, stent number, LVEF, BMI, SEAMS, GAD-2, and PHQ-2, (*p* > 0.05) (Table 4).

**Table 4.** **Characteristics of patients with ACS after PC between the low compliance–decreased and persistently high compliance groups**.

Variable		Low compliance–decreased group (n = 10)	Persistently high compliance group (n = 125)	*p*-value	Variable		Low compliance–decreased group (n = 10)	Persistently high compliance group (n = 125)	*p*-value
Gender				0.681	Occupation				0.023
	Male	9 (90.0%)	99 (79.2%)			Incumbent	7 (70.0%)	37 (29.6%)	
	Female	1 (10.0%)	26 (20.8%)			Retired	3 (30.0%)	88 (70.4%)	
Age				0.002	Daily exercise time				0.750
	<60	8 (80.0%)	37 (29.6%)			<30 min	6 (60.0%)	56 (44.8%)	
	≥60	2 (20.0%)	88 (70.4%)			30–60 min	3 (30.0%)	46 (36.8%)	
Health insurance situation				1.000		>60 min	1 (10.0%)	23 (18.4%)	
	Medical insurance	10 (100%)	119 (95.2%)		Smoking history		7 (70.0%)	55 (44.0%)	0.186
	Self-pays expenses	0 (0.0%)	6 (4.80%)		Drinking history		2 (20.0%)	39 (31.2%)	0.723
Marital status				1.000	Diabetes		2 (20.0%)	31 (24.8%)	1.000
	Married	10 (100%)	114 (91.2%)		Hypertension		5 (50.0%)	68 (54.4%)	1.000
	Unmarried/divorced/widowed	0 (0.0%)	11 (8.8%)		Stroke		0 (0.0%)	8 (6.4%)	1.000
Living situation				0.586	Medication use				
	Lives alone	1 (10.0%)	10 (8.0%)			History of aspirin	10 (100%)	91 (72.8%)	0.126
	Lives with others	9 (90.0%)	115 (92.0%)			History of ticagrelor	5 (50.0%)	42 (33.6%)	0.317
Educational status				0.778		History of clopidogrel	4 (40.0%)	48 (38.4%)	1.000
	Junior secondary and below	8 (80.0%)	88 (70.4%)			History of statins	9 (90.0%)	93 (74.4%)	0.450
	High school and above	2 (20.0%)	37 (29.6%)		Stent number				0.751
LVEF		62.70 ± 6.29	60.89 ± 7.51	0.406		<2	5 (50.0%)	55 (44.0%)	
BMI		23.59 ± 3.74	24.60 ± 3.44	0.374		≥2	5 (50.0%)	70 (56.0%)	
SEAMS		32.30 ± 5.12	32.74 ± 5.78	0.814	GAD-2		0.5 (0, 4)	1 (0, 2)	0.497
APGAR		13.40 ± 2.27	10.26 ± 2.87	0.001	PHQ-2		0 (0, 4)	0 (0, 2)	0.626

The development track category of DAPT compliance of ACS patients after PCI was used as the dependent variable (low compliance–decline group = 1; persistently high compliance group = 2). According to the clinical experience, the BMI and the categories displaying statistical significance in the univariate analysis were analyzed by multivariate logistic regression. Independent variable assignment: age: <60 years old = 1, ≥60 years old = 2; occupational status: incumbent = 1, retired = 2; the original value was used for the APGAR index and BMI being administered. Multivariate logistic regression analysis showed that age, APGAR index, and BMI affected the potential subgroup of DAPT compliance development track of ACS patients after PCI (Table 5).

**Table 5.** **Multivariate logistic regression analysis of potential categories in patients with ACS after PCI**.

	Beta	SE	Wald χ^2^	OR	95% CI	*p*-value
Aged ≥60 years	3.183	1.035	9.457	24.115	3.172~183.334	0.002
APGAR	–0.536	0.182	8.721	0.585	0.410~0.835	0.003
BMI	0.334	0.169	3.888	1.396	1.002~1.945	0.049

## 4. Discussion

### 4.1 Compliance With DAPT After PCI in Patients With ACS Presents Two Potential Categories

This study used GMM to identify two potential DAPT compliance development track categories in ACS patients after PCI: the low compliance–decreased group (7.41%) and the persistently high compliance group (92.59%). Repeated measures ANOVA was used to clarify further the significant differences in DAPT compliance of ACS patients after PCI from different groups at different time points. The statistical analysis showed significant population heterogeneity in the development track of DAPT compliance of ACS patients within 12 months after PCI.

The 7.41% incidence rate of patients calculated in the low compliance–decreased group in this study indicates that some patients may have good adherence over time in the early phase after an event, which reduces over time. A previous study [15] has shown that 4.6% of patients have poor compliance within 12 months, 4% rapidly decline, and 13.9% gradually decline during the post-discharge period following a non-fatal acute coronary syndrome or post-stroke, drug compliance; these data align with those in this study. These findings may be related to the distrust and perception of these medications among patients who experience a critical illness. The failure of patients to understand the benefits of drug treatment in time is a factor in poor compliance with cardiovascular drugs [16]. Therefore, the medical staff must be aware of these changes and initiate intensive compliance interventions for these patients before discharge and during early follow-up meetings. These include changing the perceptions of patients as to which medications are effective and preventing these patients from stopping or reducing the use of cardiovascular drugs.

The 92.59% share of patients in the persistently high compliance group in this study indicates that most patients maintain good compliance throughout the process. This may be related to the severe consequences for patients who have experienced non-fatal ACS and fear of recurrent events. These major medical events have made patients pay more attention to managing their bodies, especially managing drugs related to treating diseases.

### 4.2 Patients Aged ≥60 Years and With High BMI are More Likely to Enter the Persistently High Compliance Group

The results of this study showed that people aged ≥60 years were more likely to enter the persistently high compliance group. Cho *et al*. [17] found that participants aged ≥560 years were generally more represented in clusters with high adherence than younger participants, which was similar to the result of this study. However, Franchi *et al*. [18] reported a low adherence of older patients with chronic diseases to medication, which might be related to the fact that older patients in the community often suffer from multiple chronic diseases, with numerous concomitant medications, as well as multiple drug categories, dosage forms, and medication methods. Cao *et al*. [19] showed that younger patients had better medication compliance than older patients, and the memory and self-care ability of patients worsened with age. Rural areas and cities may also have certain differences in ages and the type of diseases, which are very important. These may be different from previous studies. Indeed, older patients are more willing to follow the advice of doctors owing to the serious consequences of the disease, and their fear of recurrent events. The medical staff needs to be aware of these changes. It is very important to identify patients with low drug compliance early and monitor drug use strategies. Moreover, individualized drug education models should be adopted for patients of differing ages and those in different disease stages. In addition, easy-to-understand vocabulary should be used. Internet plus service should be used to raise the awareness of patients regarding the importance of regular medication and the dangers of unauthorized withdrawal, and incorrect or missed drug dosages.

Populations with higher BMIs were likelier to enter the persistently high compliance group. Liu *et al*. [20] found that a higher BMI was related to good drug compliance, which was consistent with the results of this study. However, another study has also shown that obesity does not affect the drug compliance of locally advanced rectal cancer [21]. The majority of patients with high BMI values are relatively obese, which is potentially because most obese patients have chronic diseases and need to take drugs for a long time. This makes these patients more aware of their drug needs, thus improving compliance. The medical staff needs to pay more attention to these patients with less compliance in administering their medications to better institute health education on the importance of drugs, identify patients with poor compliance with medicines at an early stage, and provide incentive education plans to improve compliance.

### 4.3 Patients With High APGAR Scores are More Likely to Enter the Low Compliance–Decreased Group

This study showed that the population receiving more family care had increased access to the low compliance–decreased group. This contradicts the results of Wu *et al*. [22], which state that patients with good family functions have better medication compliance. Since most patients are highly valued by their families during hospitalization, the family care after discharge is not as good as during hospitalization, which affects their medication compliance. However, previous research has also revealed that reduced family care has a positive correlation, leading to high compliance of patients with drugs [23]. The high family care may make patients excessively rely on family members and lead to a decline in the ability of the patients to self-care. When a certain amount of assistance is missing, the self-management ability of patients will be improved to a certain extent. The medical staff should guide the family care of patients after discharge, pay attention to the compliance of patients with DAPT for home-based rehabilitation after discharge, and strengthen healthcare education for families to guide patients and drug management outside of hospitalization. Disease perception and emotional expression of familial caregivers are also very important for drug compliance [23]. The medical staff should also provide psychological education for patient family caregivers.

## 5. Conclusions

This study was designed as a longitudinal study using GMM to identify two potential categories of DAPT in ACS patients after PCI: the low compliance–decreased group and the persistently high compliance group. Age, BMI, and APGAR were important predictive factors of potential categories in developing DAPT compliance in ACS patients after PCI. According to the predictive factors, targeted compliance interventions should be formulated and implemented to help patients maintain a good development trend of compliance and improve their prognosis. There are some limitations in this study. First, this study used a single center with a small sample size, which may lead to certain selection biases. This study relies on convenience sampling, which may limit the generalizability of the findings. No longitudinal tracking evaluation of the APGAR index was conducted in this study. The potential influence of external factors such as socioeconomic status or support systems on DAPT compliance was not studied. The effect of longitudinal prediction on the development track of DAPT compliance will be explored in the future to minimize the risk of recurrent cardiovascular events.

## Availability of Data and Materials

The datasets used and analyzed during the study are available from the corresponding author upon reasonable request.
